# Intraoperative Measurement of Insertion Speed in Cochlear Implant Surgery: A Preliminary Experience with Cochlear SmartNav

**DOI:** 10.3390/audiolres14020021

**Published:** 2024-02-22

**Authors:** Stefano Concheri, Davide Brotto, Marzia Ariano, Antonio Daloiso, Valerio Maria Di Pasquale Fiasca, Flavia Sorrentino, Beatrice Coppadoro, Patrizia Trevisi, Elisabetta Zanoletti, Sebastiano Franchella

**Affiliations:** 1 Department of Neurosciences, Section of Otolaryngology, Azienda Ospedale-Università Padova, 35121 Padua, Italy; 2 Pediatric Hematology Oncology Unit, Department of Woman’s and Child’s Health, Azienda Ospedale-Università di Padova, 35122 Padua, Italy

**Keywords:** cochlear implant, insertion speed, intraoperative electrophysiological measurement, surgical training, cochlear SmartNav

## Abstract

Objectives: The objectives were to present the real-time estimated values of cochlear implant (CI) electrode insertion speed (IS) during intraoperative sessions using the Cochlear Nucleus SmartNav System to assess whether this measure affected CI outcomes and to determine whether real-time feedback assists expert surgeons in achieving slow insertion. Methods: The IS was measured in 52 consecutive patients (65 implanted ears) using the CI632 electrode. The IS values were analyzed in relation to procedure repetition over time, NRT ratio, and CI audiological outcomes. Results: The average IS was 0.64 mm/s (SD = 0.24); minimum and maximum values were 0.23 and 1.24 mm/s, respectively. The IS significantly decreased with each array insertion by the operator (*p* = 0.006), and the mean decreased by 24% between the first and last third of procedures; however, this reduction fell within the error range of SmartNav for IS (+/−0.48 mm/s). No correlation was found between IS and the NRT ratio (*p* = 0.51), pure-tone audiometry (PTA) at CI activation (*p* = 0.506), and PTA (*p* = 0.94) or word recognition score (*p* = 0.231) at last evaluation. Conclusions: The estimated IS reported by SmartNav did not result in a clinically significant reduction in insertion speed or an improvement in CI hearing outcomes. Real-time feedback of IS could potentially be used for training, but its effectiveness requires confirmation through additional studies and more accurate tools. Implementation of IS assessment in clinical practice will enable comparisons between measurement techniques and between manual and robot-assisted insertions. This will help define the optimal IS range to achieve better cochlear implant (CI) outcomes.

## 1. Introduction

The cochlear implant (CI) has proven to be effective in the rehabilitation of patients affected by severe sensorineural hearing loss, and, in selected and well-trained patients, hearing outcomes can be very close to natural function [1].

Insertion of the electrode into the scala tympani through the round window is a key surgical step that has been progressively refined and set to be as atraumatic as possible in order to achieve better outcomes [2,3]. Atraumatic insertion, along with the resulting preservation of intracochlear structures [4], has been found to increase hearing preservation, CI hearing thresholds, and speech perception capabilities [5]. To help in achieving atraumatic insertion, CI companies have developed soft electrodes that can be inserted gently into the cochlea and require less insertion force [6], reducing the risk of rupturing inner ear structures such as the basilar membrane and preventing potential scalar deviations [7].

Regarding surgical techniques, insertion speed (IS) has been investigated as a variable affecting structural preservation. Studies have shown that faster IS is associated with increased forces and risk of inner ear membrane ruptures [4,8].

To date, IS has been precisely calculated only in experimental conditions [8,9,10] or in robot-assisted insertion [11] because the precise determination in an intraoperative setting has not been feasible. In vivo IS has only been retrospectively assessed through video analysis of surgeries [4]. As a result, the optimal value for IS remains unclear [12]. Surgeons must rely on personal experience and surgical dexterity to perform slow insertions as they may not be aware of the IS they are employing.

In 2022, Cochlear^TM^ released Nucleus^®^ SmartNav System, a tool that provides CI intraoperative measurement in a wireless condition using a sterile-dressed processor that is Bluetooth-connected to an Apple iPad. There, the Cochlear app estimates IS measurement during array placement; these data are graphed in real-time. At the end of the insertion, the average IS and the total time of insertion are displayed. Then, the application performs a placement check using a proprietary transimpedance matrix algorithm [13]. The result is displayed in a scheme reporting the possible misplacement of the array, which is reported to be more frequent with pre-curved or modiolar-hugging electrodes [14]. Afterwards, stapedial reflex elicitation is optional. The final steps provide classical electrophysiological telemetries [15] such as impedance and/or neural response telemetry. When intraoperative tests are completed, the data can be exported and forwarded to the outpatient clinic, where CI will be activated and the patient followed.

The objectives of the study were as follows: (i) to report real-time intraoperative IS values provided via SmartNav; (ii) to analyze the trend of IS over time to determine if the real-time feedback affected the IS of expert surgeons; and (iii) to assess whether IS affected CI outcomes, aiming to improve our understanding of the role of this parameter.

## 2. Materials and Methods

### 2.1. Population Selection

From March 2022 to April 2023, the Cochlear^TM^ SmartNav device was used for intraoperative CI measurement in 52 consecutive patients (65 implanted ears) at the Otolaryngology Unit of the Azienda Ospedale University of Padova, that is, all patients who received Cochlear^TM^ CIs out of a total of 137 CIs. All cases were implanted with a slim modiolar electrode (CI632). For those patients who received a bilateral simultaneous cochlear implantation, each implant was analyzed independently of the contralateral.

Exclusion criteria were as follows: (i) SmartNav was not properly used for intraoperative measurement; (ii) intraoperative measurement did not include IS assessment; or (iii) cochlear malformations or other abnormalities/anomalies requiring customized array insertion were present (e.g., common cavity). 

All study procedures complied with the principles stated in the Declaration of Helsinki “Ethical Principles for Medical Research Involving ‘Human Subjects’” and with the in-house rules of the Otolaryngology Section at Padova University (Italy). Data were collected, grouped, and examined in agreement with Italian privacy and data laws. Informed consent was obtained for the procedure and the study. 

### 2.2. Patient Clinical Pathway

Adult patients (≥18 years of age) underwent pure-tone audiometry (PTA) and speech tests in the preoperative setting. Pediatric patients underwent auditory brainstem responses (ABR), behavioral or play audiometry or PTA (according to their age), and vocal tests, when feasible. The PTA score was calculated as the average hearing threshold at 0.5-1-2-4 kH. The word recognition score (WRS) was the maximum percentage of words correctly repeated by the patient out of a presented list of open-set words, regardless of the intensity of the sound.

All patients underwent preoperative computed tomography (CT), a magnetic resonance imaging (MRI) scan, and complete clinical examination.

All the interventions were performed under general anesthesia by the same team of expert surgeons (E.Z. and S.F.). A round window approach was performed in the majority of patients via mastoidectomy and posterior tympanotomy. A cochleostomy was necessary in 4 cases to facilitate array insertion. The Cochlear™ SmartNav System (version 1.1.60 and subsequent updates) was used as the intraoperative tool for IS assessment and other CI intraoperative telemetries in accordance with the User Guide (version 2.0) [16]. A wireless CP1150S surgical processor was coupled with the CI before array insertion; data are displayed on a paired Apple Inc iPad, and visible-to-the-surgeon impedance was expressed as “good” if below 30 kOhm, with an accuracy of 1 kOhm. ECAP was measured via neural response telemetry (NRT). 

After placement, the CI was activated approximately one month after surgery and patients’ follow-up was scheduled at 3, 6, and 12 months and then yearly. At each follow-up evaluation, patients underwent a CI fitting session to optimize the audiological outcome and then repeated the audiological tests.

### 2.3. Data Collection and Statistical Analysis

Age, date of surgery, side of the surgery, and unilateral or simultaneous bilateral implantation were the demographic data collected. 

During surgery, NRT values (in current levels) for each electrode and average IS were collected from the SmartNav report. NRT values were used to determine the NRT ratio, a parameter that was suggested to predict scalar change by Mittmann et al. [17]. It is defined as the average NRT value from electrodes 18–16 divided by the average NRT values from 7–5. PTA results were gathered at the initial post-operative assessment (cochlear implant activation, approximately 1-month post-surgery), with subsequent data including PTA and WRS obtained at the latest available follow-up. The auditory tests that did not investigate a single side (e.g., free-field audiometry) were therefore excluded from the statistical analysis.

IS averages between the adult and pediatric populations were analyzed using Student’s *t*-test. A linear model was employed to examine the change in IS in relation to procedure repetition. The analysis was conducted on a subgroup of procedures performed by the same surgeon (S.F.). A linear model was used to analyze the effects of IS on auditory outcomes at the first and last available follow-up. NRT ratio results were categorized by the value of 1.05, as suggested by Mittmann et al. [18]. A logistic model for dichotomous variables was used to study its relation to IS. The level of statistical significance was set at *p*-value < 0.05.

## 3. Results

A total of 52 patients (65 ears) were included in this study. There were 38 unilaterally implanted patients and 14 bilateral simultaneously implanted patients (one side did not respect the inclusion criteria and was therefore excluded). More detailed demographic data are reported in Table 1.

IS was available for all cases (*n* = 65). One adult patient had an IS of 4.31 mm/s, which is outside the range for which the accuracy of SmartNav was evaluated (0.00–4.05 mm/s, according to the User Guide) [16], so it was considered an outlier and therefore excluded in the statistical analysis. Considering the population of 64 cases, mean IS was 0.64 mm/s (SD = 0.24); minimum and maximum values were 0.23 and 1.24 mm/s respectively. The side does not appear to affect surgeon performance in terms of IS (*p* = 0.78). Similarly, the difference in IS between adult and pediatric population was not significant (*p* = 0.21). More details are available in Table 2.

**Figure 1:**
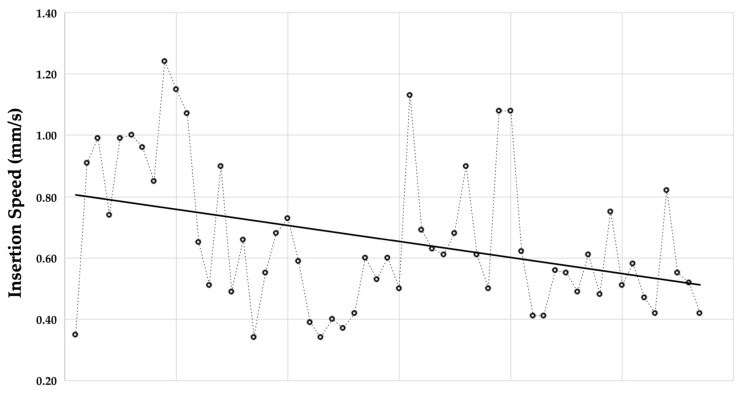
The trend of insertion speed (measured with SmartNav) with respect to insertion repetition. r = −0.366; *p* = 0.006.

The trend analysis of IS in relation to procedure repetition was performed on the subcohort of 57 CI insertions performed by the same surgeon (S.F.). The average IS for the initial third of procedures (cases 1–19) was 0.79 mm/s; whereas for the final third (cases 39–57), it decreased to 0.60 mm/s, indicating a reduction of 0.19 mm/s (24%). The overall correlation was r = −0.366 (*p* = 0.006) (see Figure 1).

The impedances were “good” for 61 of 65 positioned implants. Three of the devices had an impedance over the threshold in just one electrode of the array. The last case had four electrodes out of range. For a subgroup of 24 patients, we derived intraoperative impedances in detail: the average value was 7.51 kOhm (SD = 4.82 kOhm). 

NRT ratio was available for 59 cases; the average value was 0.89 (range 0.65–1.55). A total of 55/59 cases (93%) presented an NRT ratio ≤ 1.05. The analysis showed no impact of IS on the NRT ratio, either considering the NRT ratio as a continuous variable (*p* = 0.58) or by dichotomizing it into >1.05 and ≤1.05 values (*p* = 0.51).

The outcomes of the CI were available for a subset of the overall population—specifically, those who underwent follow-ups at our clinic. Average PTA values at CI activation were 57 dB (SD = 17 dB; n = 41). Mean duration of follow-up was 0.61 y (SD = 0.26); 31 cases had at least 6 months of follow-up and 15 cases at least 9 months of follow-up. At the last evaluation, average PTA and WRS values were 37 dB (SD = 14, n = 44) and 82% (IQR 80–100, n = 20), respectively. Audiological outcomes are detailed in Table 3.

**Figure 2:**
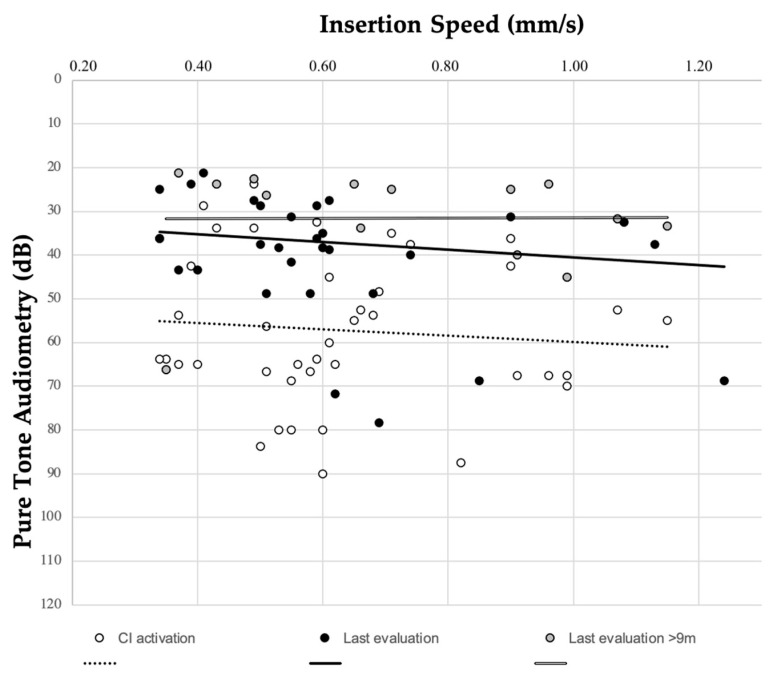
Relation between insertion speed (measured with SmartNav) and pure-tone audiometry at CI activation (r = 0.131; *p* = 0.506) and at last evaluation (r = 0.228; *p* = 0.94).

**Figure 3:**
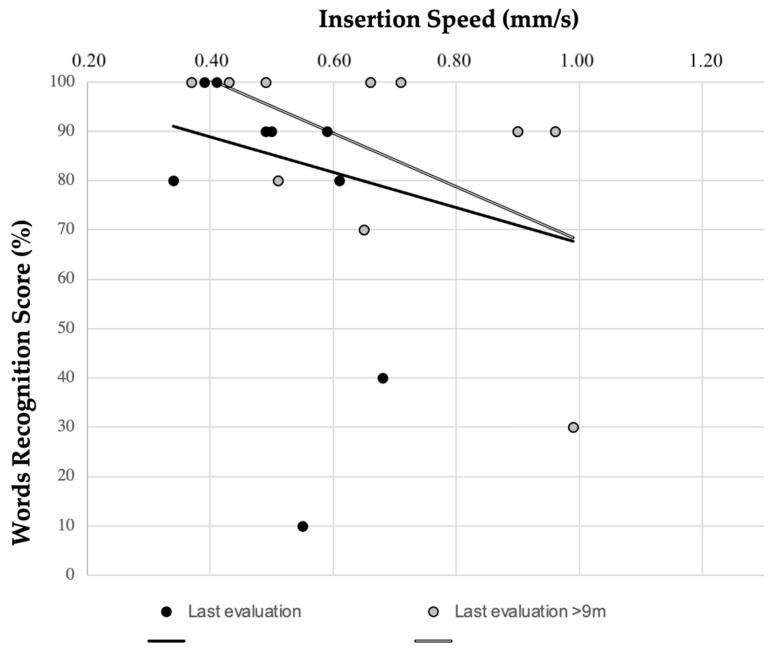
Relation between insertion speed (measured with SmartNav) and word recognition score at last evaluation (r = −0.281; *p* = 0.231).

To evaluate correlation between IS and audiological scores, free-field audiological tests were excluded. IS was found not to impact on PTA values at CI activation (r = 0.131; *p* = 0.506) or at last valuation (r = 0.228; *p* = 0.94); considering a subcohort with longer follow-up (≥9 months), the correlation was still not significant. The relation between PTA and IS is graphed in Figure 2. WRS and IS did not show any significant correlation (r = −0.281; *p* = 0.231) (see Figure 3).

## 4. Discussion

### 4.1. In Vivo Insertion Speed Measurement

Since 1993, particular attention has been paid to the parameters which could influence hearing outcomes in cochlear implantation. Lehnhardt first described the “soft surgery” cochleostomy approach, consisting in a minimal cochleostomy, inferior and anterior to the round window [2]. Since then, many technological advances have been made, allowing other variables to be introduced and considered, like the IS. Despite its prominent role, there are very few articles on the topic, and precise advice on the IS for surgeons is lacking.

The rationale of considering IS when performing a cochlear implantation is to provide atraumatic insertion of the CI array into the scala tympani. A traumatic technique could result in damage to the hearing organ, including penetration of the spiral ligament, osseous spiral lamina fractures, elevation or rupture of the basilar membrane, and array translocation into the scala media and scala vestibuli. It has been described that, consequently, intensive inflammatory and fibrotic reactions may develop, leading to unsatisfactory postoperative hearing results [3]. The existing literature examined IS in experimental settings, either involving cadaveric or synthetic bones (see Table 4); even though possible damage may be caused by an aggressive electrode insertion, there are no data on the actual function of the implant. A clear correlation between IS and the insertion forces was highlighted. Aebischer et al. [3] studied mechanical array insertions in scala tympani epoxy resin models and observed how a slower IS (total duration of 78 s with an average feed rate of 0.33 mm/s) corresponded to lower peak forces, especially if the insertion was slowed down towards the end. Kontorinis et al. [8] estimated human performances on 116 insertions in artificial scala tympani; IS ranged from 0.7–2.75 mm/s, depending on the experience of the surgeon. They concluded that high insertion speeds cause significant increases in the forces and that slow and stable IS during the insertion are recommended. Kesler et al. [19] studied human surgical performance on synthetic models. Their results indicated that the recommended value of IS (0.25 mm/s) is hard to achieve for human surgeons, since the lower limit of continuous forward insertion is 0.87 mm/s on average [19]. The study by Hugl et al. found that IS must be lowered beyond what is manually feasible (<0.1 mm/s) to significantly reduce insertion forces [11].

Despite the apparent significance of IS, studies conducted on patients providing intraoperative assessment of IS are lacking [20]. Rajan et al. retrospectively analyzed a surgical video of their patients and divided the inserted electrode length in millimeters for the total time of insertion to obtain the average IS. Their population was divided in an interventional group, whose average IS was 0.25 mm/s, and a control group, whose average IS was 1 mm/s; the ratio of hearing preservation was significantly higher and vestibular symptoms were significatively lower in the first group [4]. Snels et al. conducted a meta-analysis and observed that the rate of hearing preservation did not differ in studies reporting a slow IS compared to studies not reporting this value [20].

Given all the recommended IS values found in the literature and summarized in Table 4, the optimal IS remains unclear and appears to vary depending on the considered outcome parameter. Preservation of residual hearing is the outcome parameter considered in most of the studies, but the real benefit for implanted patients has not been definitively established. To date, IS has primarily been attained from postoperative video analysis and in vitro experiments. While performing CI insertion, surgeons are not able to understand the IS they are applying. A recommended total time of approximately 30 s is generally advised to assist the surgeon in achieving a slow insertion [12]; on the other hand, there have also been suggestions to aim for at least 2 min [21].

In the present study, we utilized SmartNav to estimate in vivo IS value. A mean value of 0.64 mm/s among a series of 64 patients was achieved. This value is lower than the classical recommendation of 1 mm/s [22], which was exceeded only in 7/64 (11%) cases in our series. The slowest IS achieved in our series was 0.23 mm/s. Consequently, the recommendation to perform an insertion at <0.25 mm/s [4,6] appears to be a realistic, albeit challenging, goal, even for freehand insertions, in contrast to what was reported by Kesler et al. [19].

The length of a Cochlear CI632 electrode is 18.4 mm from electrode tip to most proximal white marker; therefore, an IS of 0.64 mm/s corresponds to a total insertion time of 28.8 s. This value is comparable to the total time of 30 s recommended by Nguyen [12]. The total time of 2 min recommended by Jayawardena [21] corresponds to an IS of 0.15 mm/s. This value is lower than the minimum IS obtained in our series and appears to be difficult to perform manually.

All considerations made until now must be weighed based on the accuracy of SmartNav. As stated in the User Guide [16], accuracy tests conducted for IS ranging from 0.00 mm/s to 4.05 mm/s reveals a mean deviation of 0.06 mm/s and a standard deviation of the difference of 0.48 mm/s. The absolute mean deviation was not provided in the User guide [16].

**Table 4:** Summary of recommendations on insertion speed in the literature.

Author, Year	Temporal Bone Models	Type of Insertion	Calculated IS (mm/s)	Time of Insertion (s)	Recommended IS (mm/s)	Conclusion
Rau 2010 [10]	acrylic glass	M	-	20	0.5	The insertion forces appear to increase if a CI is inserted slower than 0.5 mm/s.
Kontorinis 2011 [8]	polytetrafluoroethylene (Teflon)	H	mean 1.60 range 0.7–2.75	-	“slow and stable”	High insertion speeds cause significant increase in the forces.
Rajan 2012 [4]	in vivo retrospective analysis	H	-	-	0.25	A slow electrode insertion speed reduces the occurrence of insertion resistance and increases hearing outcomes.
Pile 2013 [22]	cadaver	M	0.5–3	-	1	Mean insertion forces do not significantly reduce after insertion speeds exceed 1 mm/s.
Todt 2014 [6]	synthetic	M	-	-	0.25	Direct correlation between insertion speed and fluid pressure.
Kesler 2017 [19]	cube of bone surrogate material	H	0.86 ± 0.32	-	-	CI electrode insertion at 25 mm/s is not feasible for human operators.
Hugl 2018 [11]	f-polytetrafluoroethylene (PTFE)	M	2.8–0.045	-	“as slow as possible”	A slow electrode insertion speed reduces the occurrence of insertion resistance.
Snels 2018 [20]	human and synthetic	H, M	-	-	-	Slow insertion increases hearing preservation.
Aebischer 2021 [3]	epoxy resin	M	0.33	78	-	Slower and non-constant rate insertions decrease forces.

H, human; M, mechanical.

### 4.2. Impact on Surgeon’s Performance

Cochlear implantation is a delicate procedure that requires surgical skills [23] to slowly insert the electrode along the optimal vector, required to preserve the inner ear structures [24]. Cochlear implant surgery should receive specific training which goes beyond what is traditionally offered and performed for middle ear surgery [25].

Having real-time feedback on the IS allows for its immediate tuning, and, repetition by repetition, it may have a training effect on surgical skills. In addition, once the insertion is complete, the surgeon has immediate awareness of the average IS he employed. Simultaneous bilateral cochlear implantation, performed in our series by 24 months of age to ensure optimal audiological outcomes [26], offers the surgeon the opportunity to immediately implement adjustments based on feedback from the first side on the second side. Cochlear SmartNav aims to provide a real time IS assessment, and this value is directly visible to the surgeon. The mean IS of the last third of the iterations in our series demonstrated a 24% reduction (see Figure 1). These measures, however, fell within the margin of error for SmartNav, preventing us from drawing conclusions on the system’s efficacy. Furthermore, evaluating the training effect on an already trained surgeon may limit its effectiveness. Lastly, a control group of procedures whose IS was measured but not reported to the surgeon was not available for comparison.

Given the limitations of the tool used in this study, real-time feedback on IS seems promising but requires improvements in SmartNav accuracy or evaluation through a more accurate measurement tool. An analysis of young surgeons’ cases may be needed to determine its impact on surgical skills.

### 4.3. Insertion Speed in Relation to CI Outcomes

Slower IS has been recommended to facilitate atraumatic electrode insertion, aiming to diminish the risks of membrane rupture, scalar translocation, and tip foldover—factors that adversely impact CI outcomes. In our experience, the lowest IS achieved was 0.23 mm/s, surpassing Kontorinis’s value of 0.16 mm/s, which already corresponds to a force of 90 mN [8]. The literature reports that forces starting from 39 mN are sufficient to induce basilar membrane rupture at the basal turn [27].

Proper positioning of the CI electrode in the scala tympani has been reported to result in improved CI performances [5]. Mittmann et al. suggested that the NRT ratio may give an estimation of the intracochlear position of the electrode array. In detail, an NRT ratio > 1.05 appears to predict a scalar change, whereas an NRT ratio < 1.05 seems to be associated with the correct positioning of the array in the scala tympani (sensitivity 40.7%, specificity 92.2%, positive predictive value 73.3%, negative predictive value 74.6%) [28]. Within our series, four cases (6.7%) exhibited an NRT ratio exceeding 1.05. The literature reports a higher occurrence of scalar translocation with perimodiolar electrodes, reaching up to 51.3% as reported by O’Connell et al. [7]. This suggests a potential limitation regarding the NRT ratio. IS analysis showed no difference between these cases and the rest of the population (*p* = 0.51). This may suggest that, in our series, an increase in IS did not lead to a higher NRT ratio and, consequently, a greater risk of scalar change.

Slow IS is also supposed to determine better CI performances [12]. Their relationship with IS was determined in our series and no correlation was found, either at activation or at last evaluation. It could be inferred that minor differences in IS, as observed in our sample, seem to have no impact on CI outcomes, specifically regarding PTA or WRS.

### 4.4. Limitations and Future Developments

All the topics covered so far inevitably suffer from the limitations of this study. The accuracy of the measuring tool should be considered, even though the gold standard test used for accuracy evaluation is not declared in the User Guide [16]. No comparable techniques are currently available for straightforward intraoperative measurement of IS obtained via manual insertion. Suggested alternatives, such as intraoperative imaging or optical tracking, have limited implementation in current clinical practice. Slower and more atraumatic array placements are reported to have been achieved with robot-assisted insertion devices, currently under development [29].

Soaking the CI in saline solution before insertion (as suggested by Cochlear^TM^ before array insertion) may have an impact on an impedance-based IS measurement technique, but details about the algorithm and its sensitivity are not given in the User’s Guide [16].

**Figure 4:**
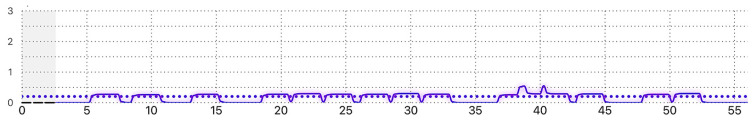
Real-time graph of insertion speed as displayed in SmartNav during electrode insertion. The presented case showed a relatively uniform IS (qualitatively assessed through graph inspection). Insertion speed (mm/s) is on the vertical axis, and time (s) is on the horizontal axis. The dotted line represents the average insertion speed.

Moreover, the sole parameter on IS provided by SmartNav is the average value. While instantaneous values are graphically displayed during insertion (see Figure 4), they are not available for consultation after the insertion, thereby impeding the analysis of IS homogeneity. Figure 4 displays an insertion with relatively uniform IS (qualitatively assessed through graph inspection), as it is commonly observed in the majority of our cases. However, some cases exhibited wider variability in IS, which outlines the role of testing the uniformity of IS. Future upgrades in SmartNav may prioritize improving the accuracy of the IS measurement tool and offering more comprehensive data, such as maximum or minimum values, homogeneity, and the time when IS = 0 (see Figure 4). This will enable a more thorough analysis of this parameter. The calculation of total time could also be more accurate and detailed. Impedance reports may include their real values, not only the range.

Lastly, analysis of series with a wider range of IS values could allow for further investigation into audiometric outcomes; however, very high IS values are not usual in most experienced CI centers.

Comparable studies analyzing intraoperative values of IS in referral to CI outcomes are lacking in the literature. In this context, the present study can be considered a first step in understanding the appropriate value or range of this parameter.

## 5. Conclusions

Insertion speed evaluated with SmartNav in a real-time intraoperative setting showed an average value of 0.64 mm/s (SD = 0.24), which complies with the total insertion time of 30 s suggested in the literature. Variation of IS does not appear to influence CI outcomes, but limitations in the accuracy of the measurement tool should be considered. Immediate feedback on IS shows potential as a training tool; however, further studies are needed to define its utility.

Implementing straightforward insertion speed (IS) assessment in clinical practice will enable comparison between different measurement techniques and between manual and robot-assisted insertion. This will help define the optimal IS range to achieve better cochlear implant (CI) outcomes. SmartNav’s role in assessing IS awaits improvements in accuracy or future studies to draw definitive conclusions.

## Figures and Tables

**Table 1:** Demographic data of the study population.

	All Patients	Adult Population	Pediatric Population
CI *n* (%)	65 (100)	20 (31)	45 (69)
Right side, *n* (%)	33 (51)	10 (50)	23 (51)
Age, y (SD)	18.2 (23.1)	49.4 (14.0)	4.4 (4.0)
≤24 m of age, *n* (%)	22 (34)	n.a.	22 (49)

CI, cochlear implant; n.a., not available.

**Table 2:** Insertion speed values in mm/s.

	All Patients (*n* = 64) *	Pediatric Population (*n* = 45)	Adult Population (*n* = 19) *	*p*-Value
Mean (SD)	0.64 (0.24) *	0.66 (0.25)	0.58 (0.21) *	0.21
Minimum value	0.23	0.34	0.23	
Maximum value	1.24 *	1.24	1.00 *	

* one value of 4.31 mm/s (adult patient) was considered an outlier and excluded in the statistical analysis.

**Table 3:** Cochlear implant outcomes in the included population.

	All Patients	Adult Population	Pediatric Population
**PTA at activation**			
n	41	27	14
dB (SD)	57 (17)	64 (14)	43 (12)
**PTA at last evaluation**			
n	44	30	14
dB (SD)	37 (14)	41 (15)	29 (8)
**WRS at last evaluation**			
n	20	7	13
% (IQR)	82 (80–100)	80 (75–95)	82 (80–100)

IQR, interquartile range; PTA, pure-tone audiometry; WRS, word recognition score.

## Data Availability

The raw data supporting the conclusions of this article will be made available by the authors on request.
